# Ultrasound-Guided Lateral Transversus Abdominis Plane (TAP) Block in Rabbits: A Cadaveric Study

**DOI:** 10.3390/ani11071953

**Published:** 2021-06-30

**Authors:** Caterina Di Bella, Luca Pennasilico, Francesco Staffieri, Federica Serino, Angela Palumbo Piccionello

**Affiliations:** 1 School of Bioscience and Veterinary Medicine, University of Camerino, 62024 Matelica, Italy; luca.pennasilico@unicam.it (L.P.); federica.serino85@gmail.com (F.S.); angela.palumbo@unicam.it (A.P.P.); 2 Department of Emergency and Organ Transplantation, Section of Veterinary Clinics and Animal Production, “Aldo Moro” University of Bari, 70010 Bari, Italy; Francesco.staffieri@uniba.it

**Keywords:** regional anaesthesia, rabbit, analgesia, transversus abdominis plane, ultrasound, spread

## Abstract

**Simple Summary:**

Small rodents and, in particular, rabbits require special attention during anaesthesia since they develop a high level of stress following painful procedures or manipulations. The transversus abdominis plane (TAP) block is a locoregional technique that has been shown to provide good perioperative analgesia for abdominal surgery, reducing the stress during recovery from anaesthesia and the need for systemic analgesic drugs. The aim of this cadaveric study was to describe a US-guided lateral TAP block technique in rabbits and evaluate the spread of the dye administered by a single injection. Our results showed that this US-guided block is applicable and easy to perform in rabbits. However, a single injection of local anaesthetic may fail to cover the sensory component of the entire abdomen. It would be interesting to evaluate the same technique in vivo but using a double injection point, as recently described in other species.

**Abstract:**

The aims of the study were to describe the ultrasonographic-guided lateral TAP block in rabbit cadavers and evaluate the spread of a lidocaine/methylene blue solution through a single fascial infiltration. The US-guided block and anatomical dissections were performed in 17 New Zealand rabbit cadavers. The probe was placed perpendicular to the column, one centimetre ventrally to the transverse processes, halfway between the iliac crest and the costal margin. External oblique (EO), internal oblique (IO), and transversus abdominis (TA) muscles were visualised, and 1 mL/kg of lidocaine 2% plus methylene blue 1% was injected. After dissection, the branches of spinal nerves stained were measured. Moreover, the percentage of length and height of the area marked were calculated. A good visualisation of the TAP was obtained in all 34 hemiabdomens. T11 nerve eminence was successfully stained in 52% of cases. T12, L1, and L2 were stained in 75%, 95%, and 100% of cases, respectively. L3 and L4 were stained in 60% and 40% of cases, respectively. The lateral TAP block with a single point of injection can be easily performed in rabbits, but it is not sufficient to cover the nerve eminences of the cranial abdomen. The two-point TAP block (lateral and subcostal) could represent a better option, particularly when large surgical incisions are required.

## 1. Introduction

The transversus abdominis plane (TAP) block is a rapidly expanding regional anaesthesia technique developed in human medicine as a result of the clinical need for a simple and efficient technique to provide good intraoperative analgesia, reduce the postoperative opioid use, increase time to first request of rescue analgesia, and provide more effective pain relief during abdominal surgeries (e.g., inguinal hernia repair, hysterectomy, caesarean delivery, laparoscopy, etc.) [1,2,3]. This locoregional technique involves the infiltration of local anaesthetic into the fascial plane between the internal oblique and transversus abdominis muscles to block the sensory components of the abdominal wall. Recently, the TAP block has been introduced in veterinary anaesthesia, both in experimental and clinical settings [4,5,6,7]. This technique involves injecting a volume of local anaesthetic into the neurovascular plane between the transversus abdominis and internal oblique muscle in order to saturate somatic afferents before they leave the TAP and provide analgesia in that anatomical area [8,9]. The lateral abdominal wall in mammals consists of three major muscle layers: the external oblique (EO), the internal oblique (IO), and the transversus abdominis (TA), along with their associated fascial sheaths [10,11]. The sensory supply for the skin, subcutaneous tissues, mammary glands, muscles, and parietal peritoneum of the cranial, lateral, and caudoventral areas of the abdominal wall is derived from the afferent branches of the ventral thoracic and lumbar nerves. These originate from the thoracolumbar spinal roots that course through the lateral abdominal wall within the fascial plane between the IO and TA muscles, termed ‘TAP plexus’ [9,12]. This site is the target of the regional block that allows obtaining lower abdominal wall analgesia [13]. Anatomical studies carried out in dogs showed that the nerves involved in TAP are the ventral branches of T11–T13 and L1–L3. These nerves are the target of the anaesthetic block in this plane [5,11]. Using the ultrasound-guided (US-guided) technique, the TAP can be correctly identified and, both the needle trajectory and injectate spread can be performed between the IO and TA muscle layers with accuracy and safety, potentially reducing the risk of block failure, accidental intraperitoneal injection, and injury to adjacent structures [14,15]. In the last years, in veterinary medicine, mainly two approaches have been described to perform the US-guided TAP block. The lateral approach is performed by positioning the US probe in a transverse orientation midway between the iliac crest and the caudal aspect of the rib cage, lateral to the midline of the abdomen [10], and the subcostal oblique approach is performed with cadavers positioned in dorsal recumbency, placing the probe caudally to the xiphoid process, parallel to the costal arch and oblique to the midline [14]. Alternative approaches with two sites of injection, one caudal to the last rib and the other cranial to the iliac crest, have been used to provide successful analgesia in dogs [12,13,14,15,16]. The spread of the volume injected and the features of these two different approaches were evaluated in dog [14,17,18], cat [7], pony [11], and calf [6] cadavers, as well as in one Canadian lynx [4], cats [19], ponies [9], and dogs [12] in vivo studies. To the authors’ knowledge, there are no studies describing the US-guided lateral TAP block in rabbits. This species requires special attention during anaesthesia since they can develop a high level of stress following painful procedures or manipulations. The use of TAP block in rabbits can reduce the stress during recovery from anaesthesia and the need for systemic analgesic drugs after abdominal surgeries. Thus, the objectives of the present study were (1) to describe a US-guided lateral TAP block technique in rabbits and (2) to assess the diffusion of a lidocaine/methylene blue solution through a single site of injection. The hypotheses were that TAP could be identified in rabbits using the US-guided approach, and the spread of the inoculated volume can be diffused to the ventral branches of T10–L3. These results would suggest a valid clinical use of the TAP block technique in this species.

## 2. Materials and Methods

This study included a total of 17 cadavers of adult New Zealand rabbits. No animals were euthanised for the purpose of this study. Moreover, as this is a cadaver study, our institution did not require ethical committee approval.

The rabbits were obtained and immediately frozen. At the time of the study, they were slowly thawed at room temperature for 48 h. All cadavers still frozen or with significant abdominal trauma or structural disease were excluded. US-guided TAP block and anatomical dissections were performed in all 34 hemiabdominal walls. Furthermore, in all cadavers, in addition to body weight and age, the length of the vertebral column from the last rib to the iliac crest (L_abdomen_; mm), the height of the hemiabdomen from the transverse processes of the spine to the linea alba (H_abdomen_; mm) and the body surface area (BSA, m^2^) were measured. The BSA was calculated using a specific formula adapted for the rabbit’s body [20] as follows:

BSA = 11.0 × BW (kg)^2/3^ (1)

### 2.1. US-Guided TAP Block

**Figure 1:**
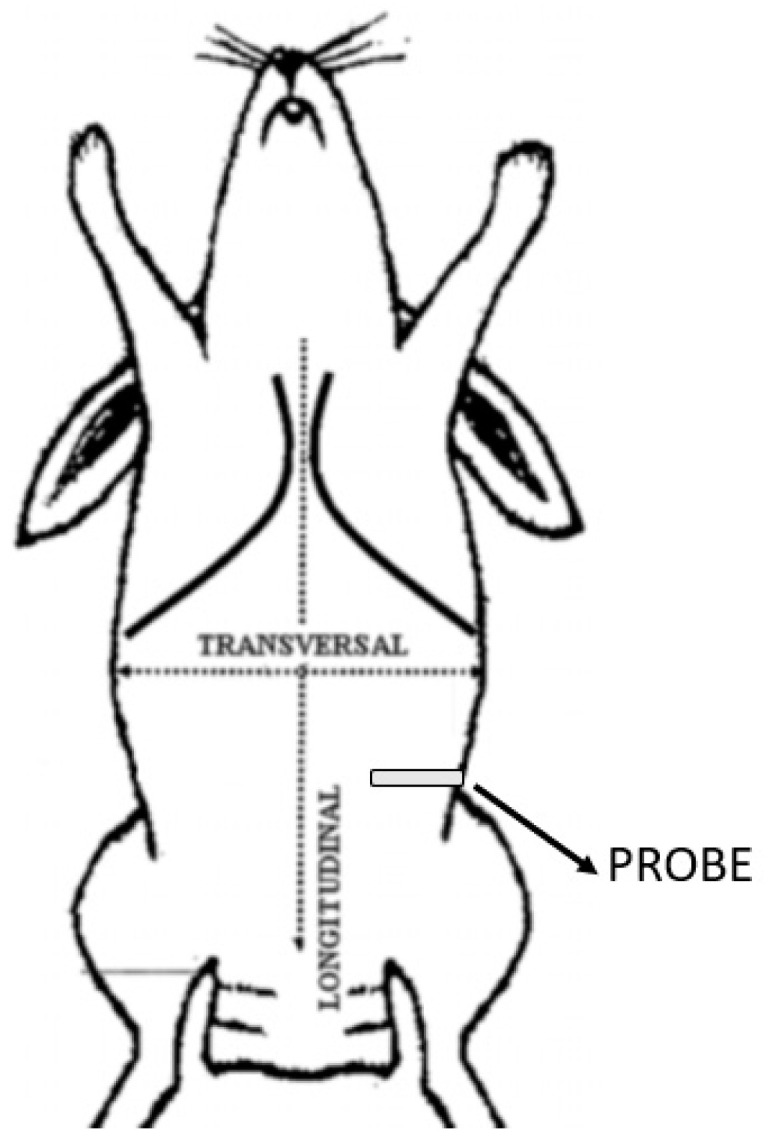
Schematic representation of the reference point corresponding to the intermediate point between the last rib and the iliac crest. The probe is positioned perpendicular to the column.

**Figure 2:**
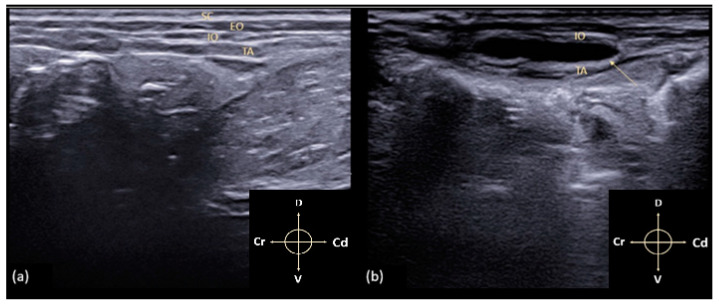
(**a**) Ultrasonographic image of the TAP anatomy in a rabbit. SC: subcutaneous tissue; EO: external abdominal oblique muscle; IO: internal abdominal oblique muscle; TA: transversus abdominis muscle; (**b**) the arrow points to a hypoechoic space between the IO and TA, corresponding to hydrodissection (i.e., separation of the muscular layers) within the TA fascia after the injection. Arrows indicate cranial (Cr), caudal (Cd), dorsal (D) and ventral (V) directions respectively.

**Figure 3:**
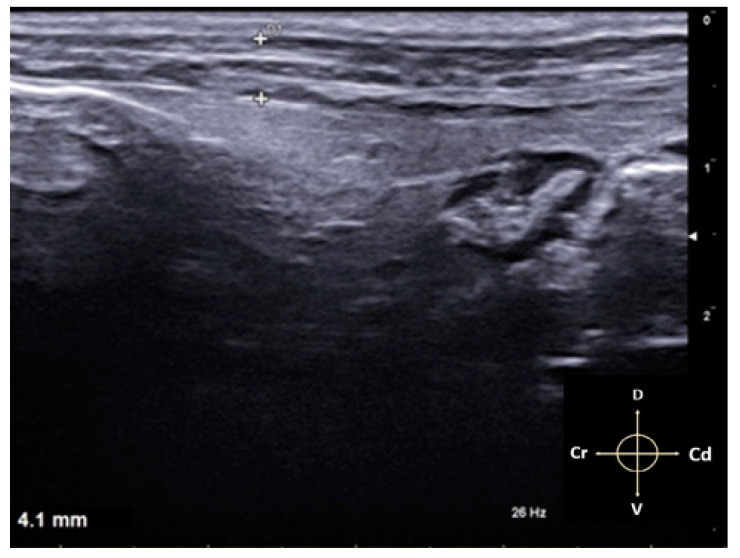
Measurements of TAP thickness in millimetres (mm). The muscle planes were measured from the SC to the peritoneum. Arrows indicate cranial (Cr), caudal (Cd), dorsal (D) and ventral (V) directions respectively.

The approach was performed with cadavers in right and left lateral recumbency. The hair of the abdomen was clipped and washed from the 10th rib to the pelvis, and alcohol was used to establish contact between the ultrasound probe and the skin. All sonographic procedures were performed by the same experienced physician using a specific US system (MyLab 9; Esaote Spa; Genova, Italy) equipped with a linear array probe (13–6 MHz; L3–11 probe; Esaote Spa; Genova, Italy). The transducer was placed transversely to the vertebral column, in the midaxillary line, halfway between the iliac crest and the costal margin (Figure 1). The view was considered satisfactory if the EO, IO, and TA muscles, peritoneum, and intraperitoneal structures were identified (Figure 2a). After measuring the depth of the TAP, a 22-gauge, 35 mm echogenic needle (Stimuplex Ultra 360 22G; B. Braun; Melsungen, Germany) was inserted through the skin, in the craniocaudal direction, cranially to the transducer, with an angle of about 30° from the body surface using an in-plane approach under direct observation, and it was advanced through the EO until the tip of the needle was resting in the fascial plane between the IO and TA muscles. Upon reaching the plane, 0.3 mL of saline was injected to confirm the correct needle position. If an uneven opacity appears within the muscle structures, an intramuscular injection was hypothesised; thus, the tip of the needle had to be repositioned. Once the needle tip was in the correct plane, the needle was held still and attached to an extension set with a syringe containing a total volume of 1 mL/kg of lidocaine 2% (Ecuphar Italia S.r.l., Milan, Italy) mixed with methylene blue dye 1% (Alcyon Spa; Rome, Italy) as a 1:1 solution. After the injection, the hydrodissection (i.e., separation of the muscular layers) within the fascia was observed as a hypoechoic space (Figure 2b). The volume of the extension set (0.5 mL) was calculated extra, and therefore, it was not necessary to flush the connection. The entire procedure was repeated on the contralateral hemiabdominal wall. The overall thickness and depth of the individual layers were measured in millimetres (mm) in all cadavers (Figure 3). Fifteen minutes after the TAP infiltration, the cadavers were dissected.

### 2.2. Anatomical Dissection

The rabbit cadavers were placed in dorsal recumbency and a sagittal incision, for removal of the skin, was made from the xiphoid process to the pubis. The skin was reflected to expose underlying structures, and the aponeurosis of the rectus and EO muscle was severed. After that, the cadavers were moved laterally and the aponeurosis of the IO muscle was dissected, starting from the linea alba, and exposing the TA muscle. The spread and the presence of dye in the correct fascial plane were evaluated by an experienced surgeon and anaesthetist, measuring the spread limits of the solution in cranial-to-caudal (CC_spread_; mm) and dorsal-to-ventral (DV_spread_; mm) directions and branches of spinal nerves stained. The nerve was considered to have been stained successfully when the dye was detected around their circumference for a length of ≥1 cm. Subsequently, the total area (mm^2^) of the local anaesthetic stained was calculated with specific software (Image-J software; ImageJ 1.45 s freeware, National Institutes of Health, Rockville, MD, USA; http://imagej.net/ImageJ) [21]. The length (L_methyl_; %) and height (H_methyl_; %) of the marked abdominal areas were calculated as a percentage of the total abdominal length and height. If the dye was found in the wrong fascial plane, no further evaluation was performed. Statistical analysis was performed using MedCalc software 9.0 (MedCalc version 9.2.10; MedCalc Software, Ostend, Belgium). The parametric data were tested for normal distribution by the Shapiro–Wilk test and are presented as mean ± standard deviation (SD).

## 3. Results

Overall, 34 abdominal walls of 17 adult New Zealand rabbits were scanned, injected, and dissected. The cadavers selected were 7 males and 10 females with an average body weight of 3.02 ± 0.37 kg and an average age of 14.42 ± 3.27 months. Mean ± SD of body surface area (BSA = 236,357.02 ± 17,998.01 mm^2^), abdominal length (L_abdomen_ = 101.61 ± 8.63 mm) and abdominal height (H_abdomen_ = 98.63 ± 9.43 mm) were calculated.

### 3.1. US-Guided TAP Block

**Figure 4:**
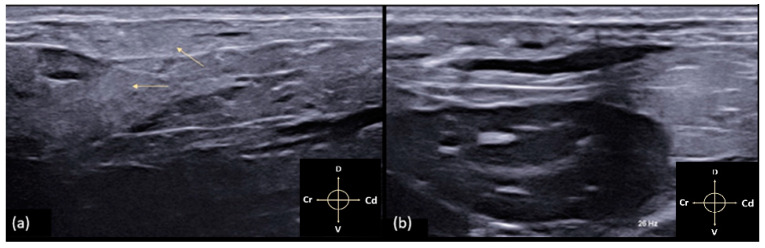
(**a**) The arrows indicate the increase in thickness and hypoechogenicity of the muscle planes due to the erroneous intramuscular infiltration of the test solution; (**b**) needle repositioning and correct inoculation of the solution. Arrows indicate cranial (Cr), caudal (Cd), dorsal (D) and ventral (V) directions respectively.

The specific reference points, previously described, allowed good visualisation of the TAP in all 34 hemiabdomens. Due to the presence of the cecum and the artifacts related to the presence of air inside it, the correct individuation of the muscular layers in the right hemiabdomen resulted in more difficulty for the operator. The EO muscle is the thinnest of the three muscle layers measured, and it appeared as a thin hypoechoic line running craniocaudally from the last rib to the pubis and dorsoventrally, from the lumbodorsal fascia to the linea alba. The IO and TA muscles are identifiable as two hypoechoic layers separated from each other and from the EO by the thick hyperechoic lines corresponding to the fascial sheaths. Below the TA muscle, the parietal peritoneum was clearly visualised as a hyperechoic linear structure separating the muscle layers from the abdominal organs. The mean overall thickness of the three muscle layers was 4.2 ± 0.3 mm. The mean and standard deviation of the individual muscle layers were EO = 1.21 ± 0.26 mm, IO = 1.33 ± 0.34 mm, and TA = 1.39 ± 0.14 mm. During the procedure, the injection site was not correctly located immediately in five hemiabdomens, and the test solution was wrongly injected intramuscularly. The needle was then repositioned, and the anaesthetic was inoculated correctly (Figure 4a,b).

### 3.2. Anatomical Dissection

**Figure 5:**
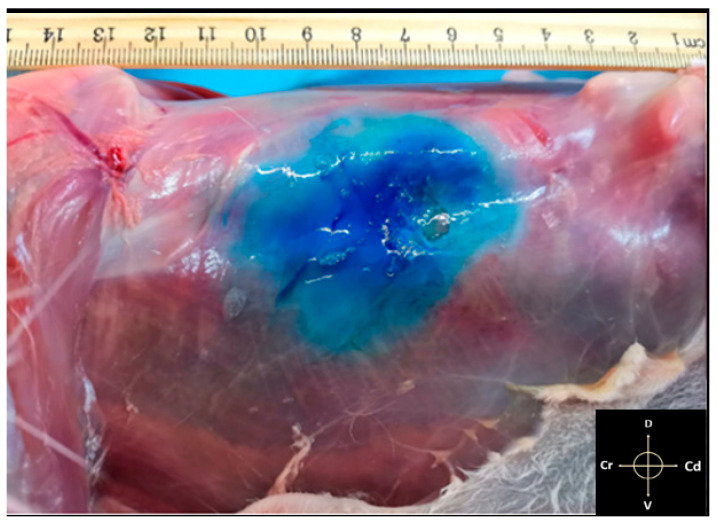
Lateral view of the abdominal wall after dissection of the skin and subcutaneous tissue. it is possible to observe the underlying tissues stained by methylene blue. The measurements from the last rib until the iliac crest were carried out. Arrows indicate cranial (Cr), caudal (Cd), dorsal (D) and ventral (V) directions respectively.

**Figure 6:**
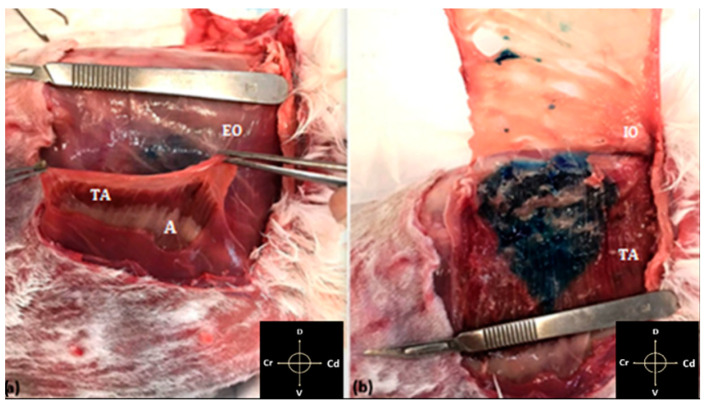
(**a**) Removal of the external and internal oblique muscles. It is possible to observe the aponeurotic fascia (A) of the underlying rectus abdominis and ventral portion of the transverse abdominis muscles (TA); (**b**) complete dissection of the internal (IO) and external oblique (EO) muscles. It is possible to observe the characteristic orientation of the muscle fibres of the transversus of the abdomen. The methylene blue has been correctly inoculated, and the stained muscle tissue is visible. Arrows indicate cranial (Cr), caudal (Cd), dorsal (D) and ventral (V) directions respectively.

In all dissected cadavers, the methylene dye was visible within the neurovascular plane between the TA and IO muscles, with small needle puncture traces between the skin and the EO muscle (Figure 5). The three layers of the abdominal wall were successfully dissected in 30 hemiabdomens (Figure 6a,b). In the remaining four, probably due to frostbite, it was not possible to separate the IO from the TA muscles. Furthermore, in one of these cadavers, the cecum was punctured; therefore, the evaluation of these four hemiabdomens was not completed. The most cranial spread of the methylene blue was detected at T11 nervous eminence, which was successfully stained in 52% of the cases. T12, L1, and L2 were successfully stained in 75%, 95%, and 100% of cases, respectively. The L3 and L4 nervous eminence were successfully stained in 60% and 40% of the cases, respectively. None of the nerves cranial to T11 and caudal to L4 were marked by a TAP injection. The mean ± SD of craniocaudal (CC_spread_) and dorsoventral (DV_spread_) spread, the percentage of the total length (L_methyl_) and the height (H_methyl_) of hemiabdomens stained, and the average of the area marked are described in Table 1.

## 4. Discussion

Based on literature searches, this is the first study in which the lateral TAP block is performed in rabbit cadavers. In this study, we found that the ultrasonographic identification of the TAP was feasible and easy to perform in rabbits. Moreover, the results obtained after anatomical dissection show that a single lateral inoculation with 1 mL/kg of anaesthetic is not sufficient to cover the sensitive branches of the cranial abdomen.

The ultrasound anatomy of TAP in rabbits is similar to that previously reported in cats [7,19]. Furthermore, the thickness of the individual muscle layers is very similar [7]. The knowledge of the thickness of the muscle layers and of the individual layers could be useful in clinical practice to facilitate the recognition of anatomical structures and perceive the depth of the fascial plane to be reached. However, we must consider that the ultrasonographic visualisation of the anatomical planes was more difficult in rabbits because of the large and superficial cecum and colon. For this reason, the authors’ opinion is that the use of ultrasonography and the good experience of competent personnel are mandatory for the correct execution of the technique. Another important difference to consider is that, in rabbits, the thoracic nerve eminences are 12 (T1–T12) and not 13, as in other species (e.g., dog, cat). This factor was then considered in the analysis of the cranial spread. A volume of 1 mL/kg of lidocaine and methylene blue solution was injected to identify the dye in the neurovascular plane. This volume was chosen based on previous scientific studies [22]. Bruggink et al. have shown that larger volumes of local anaesthetic have a greater diffusion in the plane, and a solution of 1 mL/kg is able to cover up to four nerve roots in the TAP [22]. However, in a clinical setting, the choice of the volume injected is influenced by the need not to exceed the toxic dose of the local anaesthetic [7]. Based on our results, the most cranial diffusion of methylene blue was detected at T11 nerve eminence (successfully stained in 52% of cases), while nerve eminences of L3 and L4 were stained in 60% and 40% of cases, respectively. This suggests that, despite the high volume used, in a clinical context, a single injection of local anaesthetic would not be able to adequately cover all abdominal sensory branches in the TAP. Moreover, in agreement with the results of Zoff et al. and Romano et al. [16,23], we found a significant dorsoventral diffusion (44.48 ± 9.26 mm). This showed that the use of large volumes probably does not improve the craniocaudal spread (48.91 ± 7.56 mm) but induces diffusion of local anaesthetic evenly over the entire abdominal wall. It is the authors’ opinion that it could be useful in this species, as already demonstrated in the cat [7] and dog [16] cadavers, to use a two-point TAP block (subcostal and lateral) to obtain a better cranial distribution of the local anaesthetic and to reduce the total volume injected.

Compared to classic neuraxial techniques, fascial plane blocks are considered at low risk of nerve injury and relatively easy to perform; however, it should be emphasised that the TAP block only provides somatic analgesia (from the ventrolateral skin of the abdomen to the peritoneum); therefore, during surgery involving visceral pain, systemic drugs are needed to achieve adequate analgesia [15,23]. Either way, according to the current literature in human and veterinary medicine, the TAP block has been demonstrated to produce great results as part of a multimodal approach in the management of perioperative abdominal pain [15,16,17,24].

Some limitations need to be considered in this study. The technique was performed on cadavers gradually thawed at room temperature. Although the persistence of the freezing state was considered among the exclusion factors, the execution of this technique in cadavers may have influenced the diffusion of the solution (different tissue integrity, body temperature, and blood and lymphatic perfusion). Therefore, the diffusion of the solution, the ultrasound execution, and the extent of the sensory block should be evaluated in vivo to evaluate its efficacy. It should also be considered that the presence of significant meteorism developed postmortem in the cecum makes it more difficult to perform the US technique due to the presence of artifacts. Another limit to consider is the anatomical differences between the rabbit and other species previously studied. It would be useful to perform an anatomical study of the TAP also in this species. Moreover, in this study, we did not consider the minimum volume that can be administered, but we chose to use a high volume to be injected (1 mL/kg) to perform a preliminary evaluation of the technique and its effectiveness in this species. In the clinical setting, in vivo, it is clear that the maximum toxic dose must be evaluated and the doses considerably reduced.

## 5. Conclusions

The results obtained in this cadaveric study indicate that the TAP block performed with a single lateral injection can be easily performed in rabbits, but it is probably not sufficient to cover the nerve eminences of the cranial abdomen. Based on the results observed from this study and the findings from previous studies in other species [7,16], the two-point TAP block (lateral and subcostal) could represent a better option to provide adequate analgesia during abdominal surgery in this species, particularly when a large surgical incision is required. However, to evaluate the real analgesic efficacy, in vivo studies are necessary.

## Figures and Tables

**Table 1:** Height and length (mean ± SD) of all hemiabdomens (H_abdomen_, L_abdomen_) and of stained area (DV_spread_; CC_spread_) in millimetres (mm). The percentage of height (H_methyl_, %) and length (L_methyl_, %) of the stained area and the overall area of the spread (Area stained, mm^2^) were also calculated and reported in the table.

Measurement	L_abdomen_ (mm)	H_abdomen_ (mm)	CC_spread_ (mm)	DV_spread_ (mm)	L_methyl_ (%)	H_methyl_ (%)	Area Stained (mm^2^)
MEAN	101.615	98.630	48.916	44.485	48.17	43.41	2515
SD	8.63	9.43	7.56	9.26	6.78	8.98	595.05

## Data Availability

The data presented in this study are available on request from the corresponding author.
